# Dietary *Bacillus coagulans* supplementation improves liver health and modulates hepatic metabolic profiles in juvenile fourfinger threadfin (*Eleutheronema tetradactylum*)

**DOI:** 10.3389/fmicb.2026.1909946

**Published:** 2026-08-26

**Authors:** Jing Li, Nuo Chen, An-Na Zheng, Shui-Ping He, Wei-Bin Liu, Jing-Heng Lu, Min-Xuan Jin, Lin-Juan Wang, Hui-Juan Zhang, Evodia Moses Mkulo, Bao-Gui Tang, Hui Zhou, Bei Wang, Jian-Sheng Huang, Zhong-Liang Wang

**Affiliations:** 1 College of Fisheries, Guangdong Ocean University, Zhanjiang, Guangdong, China; 2 Guangdong Marine Fish Science and Technology Innovation Center, Zhanjiang, Guangdong, China; 3 Guangdong Provincial Key Laboratory of Aquatic Animal Disease Control and Healthy Culture, Zhanjiang, Guangdong, China; 4 Agro-Tech Extension Center of Guangdong Province, Guangzhou, China

**Keywords:** *Bacillus coagulans*, *Eleutheronema tetradactylum*, hepatic metabolism, metabolomics, probiotics

## Abstract

**Introduction:**

Dietary supplementation with probiotics has emerged as a promising strategy to enhance the health and productivity of farmed fish. However, the systemic metabolic effects of probiotics on the liver of marine aquaculture species, particularly the underlying molecular mechanisms, remain insufficiently explored. This study employed histopathological examination, biochemical assays, and non-targeted liver metabolomics to systematically evaluate the effects of dietary *Bacillus coagulans* on hepatic morphology, oxidative status, and metabolic profiles, and to elucidate the potential metabolic regulatory mechanisms underlying its protective action in fourfinger threadfin (*Eleutheronema tetradactylum*).

**Methods:**

Juvenile *E. tetradactylum* were fed either a control diet or a diet supplemented with 1 × 10⁸ CFU/g *B. coagulans*. At the end of the eight-week feeding trial, liver samples were collected to evaluated the effects of dietary *B. coagulans* on the hepatic morphology, oxidative status, and metabolic profiles.

**Results:**

*B. coagulans* supplementation improved hepatic tissue architecture, characterized by a more orderly cellular arrangement and reduced fibrosis. ALT activity was elevated in the *B. coagulans* group, indicating enhanced amino acid metabolism rather than hepatocellular injury. Activities of AKP, LZM, CAT, and SOD remained at healthy baseline levels with no significant intergroup differences, while MDA showed a non-significant downward trend. Metabolomics revealed 43 altered hepatic metabolites, with pathway enrichment indicating upregulated amino acid turnover, enhanced lipid metabolism (including cholesterol clearance), accelerated tricarboxylic acid cycle (increased isocitric acid), and elevated xanthine, a potent endogenous antioxidant.

**Conclusion:**

Collectively, these findings demonstrated that *B. coagulans* supplementation safely optimizes hepatic metabolic flux and structural integrity, highlighting its potential as a highly effective functional additive to promote metabolic homeostasis in the aquaculture of *E. tetradactylum*.

## 1 Introduction

The fourfinger threadfin (*Eleutheronema tetradactylum*), a warm-water, euryhaline fish that inhabits freshwater or brackish water at depths up to 30 meters, has gained attention for its rapid growth rate, short cultivation cycle, and high market value (Xuan and Wang, 2023; Jin et al., 2024). Given these advantages, *E. tetradactylum* has been designated by the Food and Agriculture Organization as a priority species for marine aquaculture promotion, demonstrating extremely promising prospects for aquaculture development (Chen et al., 2025). However, as the scale of its aquaculture expands, the physiological health of this species has been challenged by high-density farming practices.

Under high-density farming stressors, lipid metabolism serves as a key regulatory pathway, providing essential fatty acids and energy while supporting immune responses (Lee et al., 2018) The liver is a critical metabolic organ in fish, performing vital functions such as detoxification, glycogen storage, and the synthesis of secretory proteins (Bechmann et al., 2012). The health condition of the liver directly determines the growth rate and disease resistance of fish. In aquaculture systems, the liver dysfunction in farmed fish can be easily induced by a combination of factors including elevated ammonia-nitrogen concentration caused by excessive stocking density, improper feed nutrient ratios, and the overuse of medications (Zhang et al., 2019). When liver function is impaired, the activity of key metabolic enzyme systems in the fish changes, and the structural integrity of hepatic tissue is also compromised. At the same time, the immune defenses were weakened by metabolic dysfunction in the liver, making farmed fish more susceptible to large-scale outbreaks of infectious diseases and causing severe economic losses to the aquaculture industry.

*Bacillus coagulans* is the probiotic strain that has attracted significant attention in recent years. Due to its non-toxic and non-pathogenic nature, it shows great potential for application in the aquaculture. Although *B. coagulans* cannot colonize the gut for extended periods, its metabolic products have positive effects on the immune activity, disease resistance, and growth of various aquatic animals (Konuray and Erginkaya, 2018; Hu et al., 2024; Su et al., 2025). Numerous studies indicate that *B. coagulans* plays a multifaceted positive role in protecting the liver of aquatic animals. In a study on *Cyprinus carpio*, Khalil et al. found that the combined use of *B. coagulans* and a type of algae could increase growth rates and feed utilization in carp, enhance their nonspecific immune capacity, and maintain intestinal and liver health (Khalil et al., 2026). Similarly, dietary supplementation with *B. coagulans* in rainbow trout (*Oncorhynchus mykiss*) has been shown to significantly improve growth parameters and reduced feed conversion ratios, while also enhancing antioxidant enzyme activity and alleviating liver damage caused by herbicides (Raeeszadeh et al., 2025).

While recent evidence has demonstrated that dietary *B. coagulans* supplementation effectively promotes growth performance and improves intestinal structural integrity in juvenile *E. tetradactylum* (Zheng et al., 2025), there has been a lack of systematic and in-depth analysis of the underlying molecular mechanisms by which *B. coagulans* affects hepatic metabolism. Given the critical role of the gut-liver axis in nutrient partitioning and immune regulation, it is essential to understand the hepatic metabolic responses to probiotic interventions. In this study, using juvenile *E. tetradactylum* as the experimental subject, we employed histopathological examination, biochemical assays, and non-targeted liver metabolomics analysis to systematically investigate the protective effects of dietary *B. coagulans* supplementation on the liver of *E. tetradactylum* and elucidate its potential metabolic regulatory mechanisms. The aim is to provide a theoretical basis and technical support for the scientific application of *B. coagulans* in the healthy aquaculture of *E. tetradactylum*.

## 2 Materials and methods

### 2.1 Fish rearing and experimental design

Juvenile *E. tetradactylum* were purchased from a local commercial fish company. The feed was purchased from Weifang Santong Biological Engineering Co., Ltd. (Weifang, China), with guaranteed nutritional composition of crude protein (content ≥ 55%), crude fat (content ≥ 8%), crude fiber (content ≤ 3%), and crude ash (content ≤ 16%). The *B. coagulans* T-21 strain was supplied by Kunming Aikete Biotechnology Co., Ltd. (Kunming, China).

*B. coagulans* T-21 strain was inoculated into MRS broth for enrichment culture. After 48 h, the culture was centrifuged to collect the pellet, which was washed three times with sterile phosphate-buffered saline (PBS) and finally resuspended in PBS to 1 × 10^9^ CFU/mL. The cultured *B. coagulans* was serially diluted and evenly sprayed onto the feed surface to a concentration of 1 × 10^8^ CFU/g. After dehydration, the feed was stored at −20 °C for future use.

Prior to the trial, 420 juvenile *E. tetradactylum*, healthy and uniform in size, were randomly divided into two groups which contained three replicates with 70 juveniles in each replicate, and acclimated in the tanks for 1 week. In the formal experiment with a span of 8 weeks, the juvenile fish in the control group were regularly fed three times each day at 8:00, 12:00 and 17:00, while fish in the *B. coagulans* group were fed the *B. coagulans* supplemented diet. Water quality was monitored regularly to ensure the following parameters: temperature at 27–29.5 °C, salinity at 27–30, dissolved oxygen above 6 mg/L, pH 7.8–8.0, and ammonia nitrogen below 0.3 mg/L. The experiment was conducted in an indoor recirculating aquaculture system with filtered seawater.

### 2.2 Sample collection

After the feeding trial, the juvenile fish were randomly selected from each tank and sampled. The whole liver was collected and frozen in liquid nitrogen, and stored at −80 °C.

### 2.3 Hepatic histological examination

Liver tissues were fixed in 4% paraformaldehyde, embedded in paraffin, sectioned at 5–7 μm, deparaffinized with xylene, and stained with hematoxylin and eosin (H&E) before observation under a Nikon 80i optical microscope.

### 2.4 Hepatic enzyme activity assay

Immune parameters (AKP, LZM, and ALT) and antioxidant stress parameters (CAT, MDA, and SOD) were determined in liver tissue homogenates using commercial kits obtained from Nanjing Jiancheng Institute, following to the manufacturer’s instructions.

### 2.5 Hepatic metabolomics sequencing analysis

#### 2.5.1 Sample treatment

After thawing slowly at 4 °C, approximately 100 mg of liver tissue was sectioned on dry ice and transferred into a 2 mL Eppendorf tube. For metabolite extraction, 1 mL of pre-chilled mixed solvent (methanol: acetonitrile: water in a 2:2:1 volume ratio) was added. The samples were homogenized using an MP-type homogenizer (set program parameters to 24 cycles × 2, at a speed of 6.0 m/s, with each cycle lasting 20 s, repeated 3 times).

After grinding, the samples underwent two 30-min cycles of low-temperature ultrasonic treatment, were allowed to settle at −20 °C for 60 min, and then centrifuged for 15 min (14,000 g, 4 °C). The supernatant was collected and aliquoted into 900 μL/tube. The samples were vacuum freeze-dried, and the resulted powder was stored at −80 °C for subsequent analysis. Prior to mass spectrometry analysis, 100 μL of resuspension solution (acetonitrile: water in a 1:1 volume ratio) was added to the sample powder, and thoroughly vortexed.

Quality control (QC) samples prepared by mixing equal volumes of all test samples, were used to assess the condition of the instrument as well as the equilibration and stability of the chromatography-mass spectrometry system throughout the entire experimental process.

#### 2.5.2 LC–MS/MS analysis

Analysis was performed using an UHPLC (1,290 Infinity LC, Agilent Technologies) coupled to a quadrupole time-of-flight (AB Sciex TripleTOF 6,600) in Shanghai Applied Protein Technology Co., Ltd. Three samples in each group were used for analysis.

The chromatographic conditions were set as follows. In both electrospray ionization (ESI) positive and negative modes, the mobile phase contained A = 25 mM ammonium acetate and 25 mM ammonium hydroxide in water and B = acetonitrile. The gradient was 85% B for 1 min and was linearly reduced to 65% in 11 min, and then was reduced to 40% in 0.1 min and kept for 4 min, and then increased to 95% in 0.1 min, with a 5 min re-equilibration period employed. Samples were injected via an automatic sampler at 4 °C, with a 2 μL injection volume and a flow rate of 0.5 mL/min.

Mass spectrometry analysis was performed using ESI source with the following parameters: Ion Source Gas 1 (Gas1) and Ion Source Gas 2 (Gas2) both set to 60 psi, Curtain Gas (CUR) at 30 psi, ion source temperature at 600 °C, and ion spray voltage (ISVF) at ± 5,500 V (dual-mode scanning for both positive and negative ions). The time-of-flight mass spectrometry (TOF MS) scan range was m/z 60–1,000 Da and accumulation time was 0.20 s/spectrum, while the secondary mass spectrometry scan ranged m/z 25–1,000 Da with an accumulation time of 0.05 s/spectrum. Secondary mass spectrometry data were acquired under information-dependent acquisition (IDA) mode, set to high-sensitivity mode, with a declustering potential (DP) of ± 60 V and a collision energy of 35 ± 15 eV. Dynamic exclusion was enabled to exclude isotope peaks within 4 Da, monitoring 10 candidate per cycle. QC samples were inserted into the analysis queue in random order to monitor instrument status and data reliability.

#### 2.5.3 LC–MS/MS data analysis

The raw instrument data was converted to the MzML standard format using ProteoWizard software (version 3.0.6428), followed by chromatographic peak alignment, retention time correction, and peak area integration via the XCMS online platform (version 3.7.1). XCMS parameters were set as follows: the centWave algorithm was used with a mass deviation tolerance of 10 ppm during the peak detection stage, a peak width range of 10–60 scan points, and a signal intensity pre-filtering threshold of 10–100. During the peak grouping stage, the bandwidth was set to 5, the mass window to 0.025, and the minimum presence ratio to 0.5.

Three-tier quality control was performed on the XCMS output data: (1) In the metabolite annotation stage, structural identification was performed via matching against a standard spectral library; (2) In the data cleaning stage, null value filtering (removing ion peaks with > 50% missing values), KNN algorithm-based imputation of missing values, and RSD filtering (retaining feature peaks with a relative standard deviation ≤ 50%) were performed; (3) The resulting high-quality dataset was used for multivariate statistical analysis. This processing workflow effectively reduces the impact of technical variability on metabolomics analysis.

### 2.6 Statistical analysis

SPSS24.0 was used to analyze the samples by independent samples t-test. Data were expressed as mean ± SEM, and *p* < 0.05 was considered statistically significant.

## 3 Results

### 3.1 Improvement of hepatic histology by *B. coagulans*

**Figure 1:**
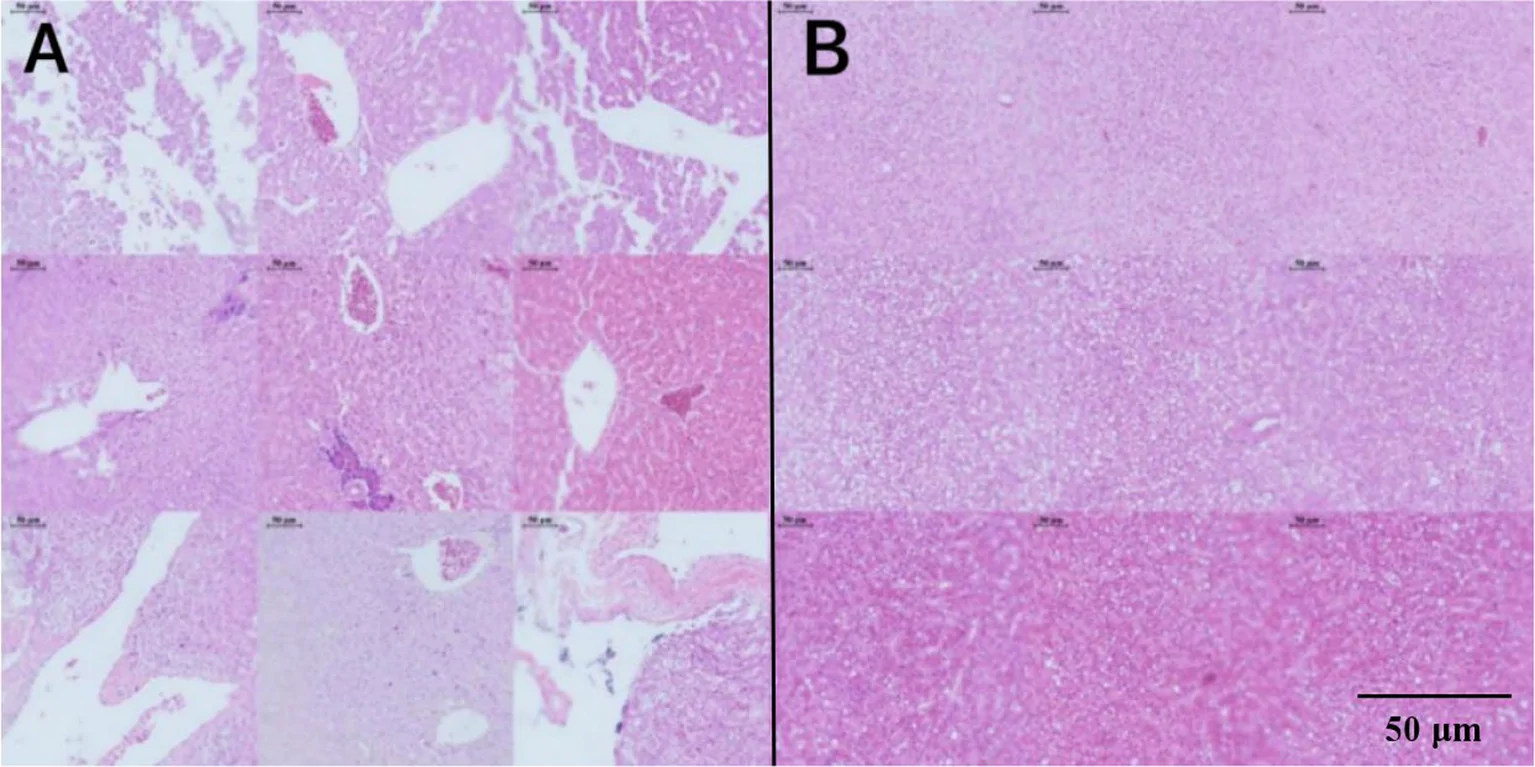
Effects of *B. coagulans* on the histological structure of the liver in juvenile *E. tetradactylum* (H&E staining). **(A)** The control group. **(B)** The *B. coagulans* group. The control group was fed a regular diet daily while the *B. coagulans* group was fed a regular diet containing 1 × 10^8^ CFU/g *B. coagulans*. Scale bar, 50 μm.

At the end of the eight-week feeding trial, representative liver tissue sections from the control group and *B. coagulans* group were shown in Figures 1A,B, respectively. The liver tissue in the control group exhibited a loose and disorganized arrangement in general. Compared with the control group, the hepatocytes in the *B. coagulans* group displayed a tighter and more orderly arrangement. There was a trend toward reduced pathological changes and fibrosis around the central vein (Figure 1).

### 3.2 Effects of *B. coagulans* on hepatic immune enzyme activities

**Figure 2:**
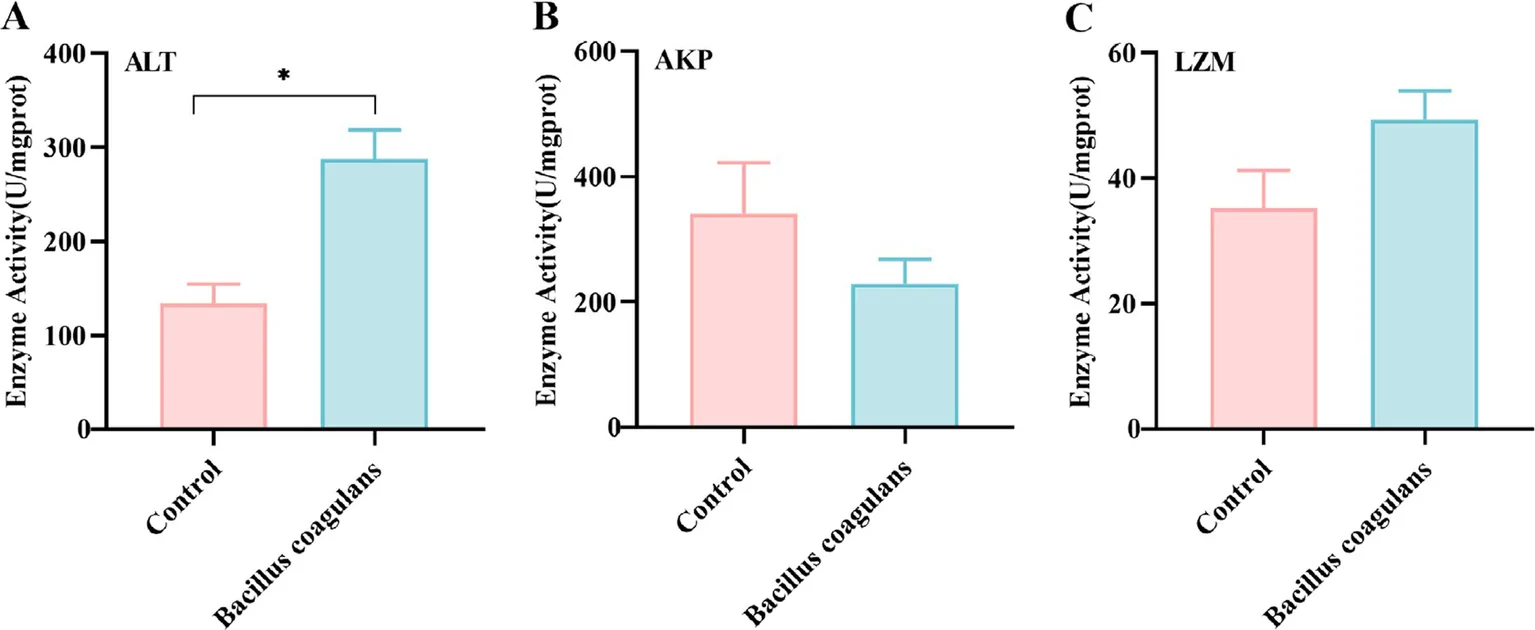
Effects of the *B. coagulans* on hepatic immune enzyme activities in juvenile *E. tetradactylum*. **(A)** Alanine aminotransferase (ALT). **(B)** Alkaline phosphatase (AKP). **(C)** Lysozyme (LZM). The control group was fed a regular diet daily while the *B. coagulans* group was fed a regular diet containing 1 × 10^8^ CFU/g *B. coagulans*. Values represent mean ± SEM (*n* = 3). Statistical analyses were performed using independent samples *t* tests. The single asterisk (*, *p* < 0.05) indicates the statistical significance compared with the control group.

The effects of *B. coagulans* on hepatic immune enzyme activities of juvenile *E. tetradactylum* were shown in Figure 2. All indicators in the experimental group presented changes compared to the control group. Among these, the activity of alanine aminotransferase (ALT) exhibited a significant increase in the *B. coagulans* group. Additionally, the activity of alkaline phosphatase (AKP) and lysozyme (LZM) slightly decreased and increased respectively, though neither change was statistically significant.

### 3.3 Effects of *B. coagulans* on antioxidant enzymes and oxidative stress marker in the liver

**Figure 3:**
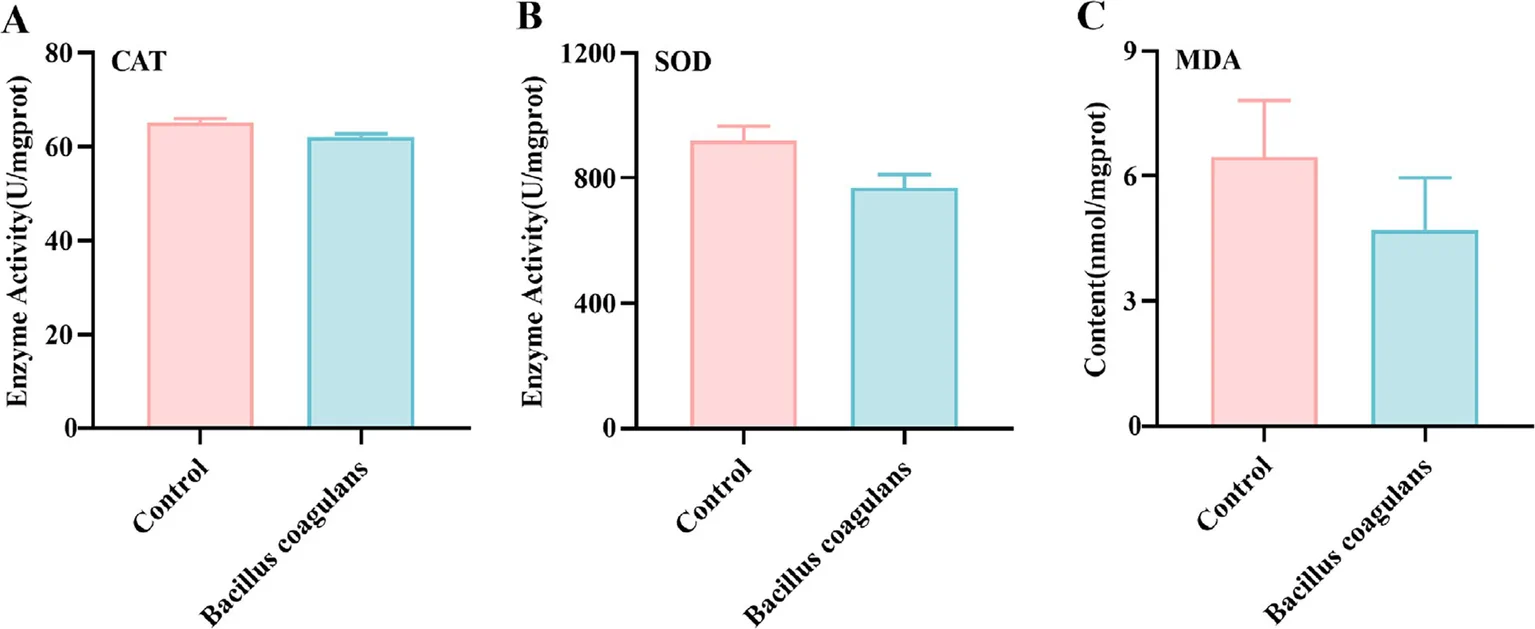
Effects of *B. coagulans* on hepatic antioxidant enzyme activities and MDA level in juvenile *E. tetradactylum*. **(A)** Catalase (CAT); **(B)** Superoxide dismutase (SOD); **(C)** Malondialdehyde (MDA). The control group was fed a regular diet daily while the *B. coagulans* group was fed a regular diet containing 1 × 10^8^ CFU/g *B. coagulans*. Values represent mean ± SEM (*n* = 3). Statistical analyses were performed using independent *t* tests.

The effects of *B. coagulans* on the antioxidant capacity of the livers of juvenile *E. tetradactylum* were presented in Figure 3. Compared with the control group, no statistical significance was observed in the activities of antioxidant enzymes catalase (CAT), superoxide dismutase (SOD), or the level of malondialdehyde (MDA) in the *B. coagulans* group (*p* > 0.05).

### 3.4 Metabolic analysis in liver

#### 3.4.1 OPLS-DA model construction

**Figure 4:**
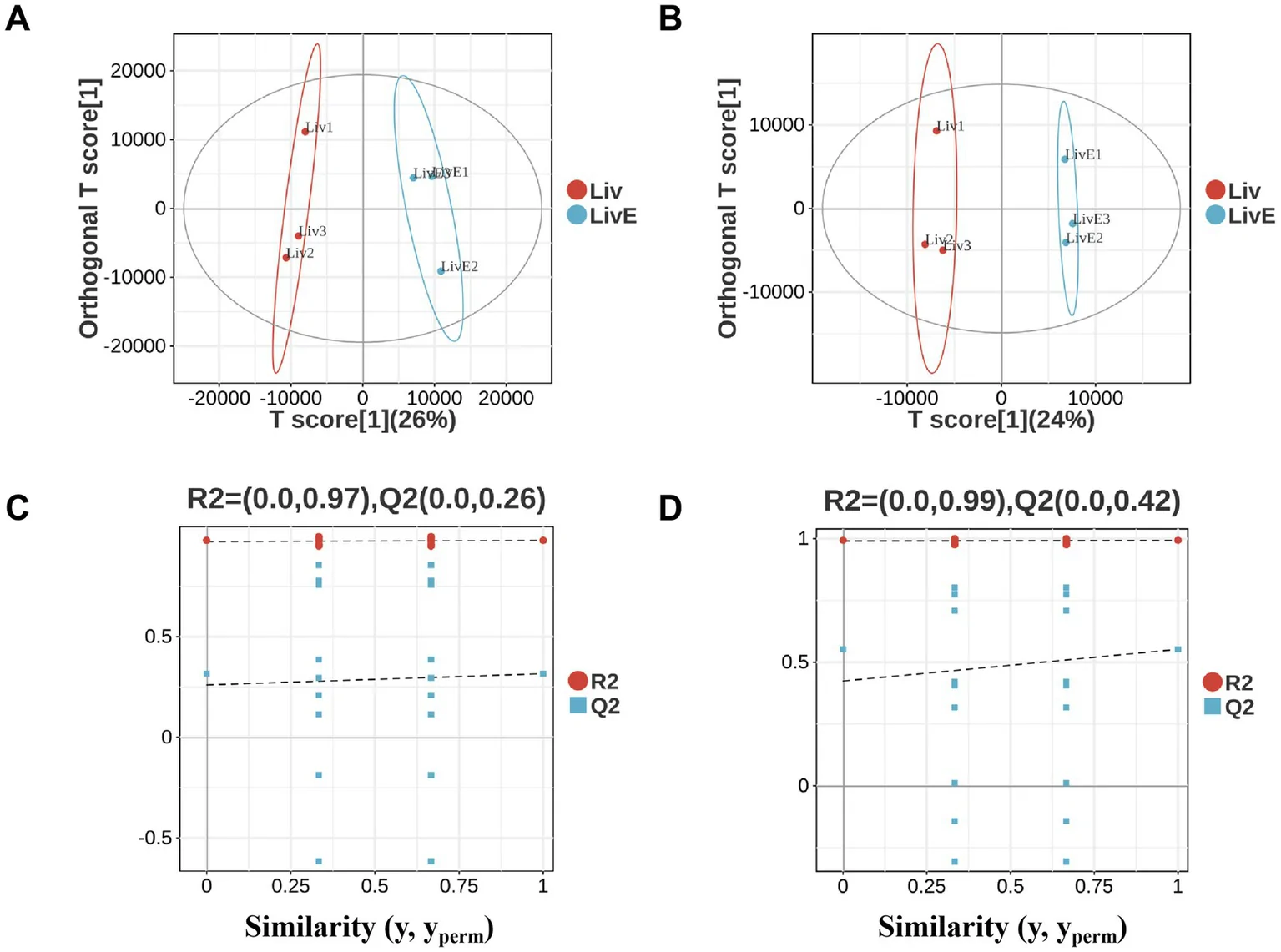
OPLS-DA score charts and model validation plots for liver metabolomics. **(A)** OPLS-DA score chart in positive ion scanning mode. **(B)** OPLS-DA score chart in negative ion scanning mode. **(C)** Model validation plot in positive ion scanning mode. **(D)** Model validation plot in negative ion scanning mode. Liv represents the control group. LivE represents the *B. coagulans* group. OPLS-DA: Orthogonal partial least squares discriminant analysis.

To investigate the differences in metabolites between the *B. coagulans* group and the control group, orthogonal partial least squares discriminant analysis (OPLS-DA) was used to perform intergroup comparisons. The OPLS-DA score plots illustrated that, under both positive and negative ion scanning modes, the metabolite distributions of the control group and the *B. coagulans* group significantly separated, and the metabolic profiles of the two groups exhibited significant differences. To further validate the reliability of the model, a cross-validation test was conducted. The results demonstrated that in the positive ion mode, the OPLS-DA model had an R^2^ of 0.97 and a Q^2^ of 0.26, while the model had an R^2^ of 0.99 and a Q^2^ of 0.42 in the negative ion mode (Figure 4).

#### 3.4.2 Key differential metabolites

**Figure 5:**
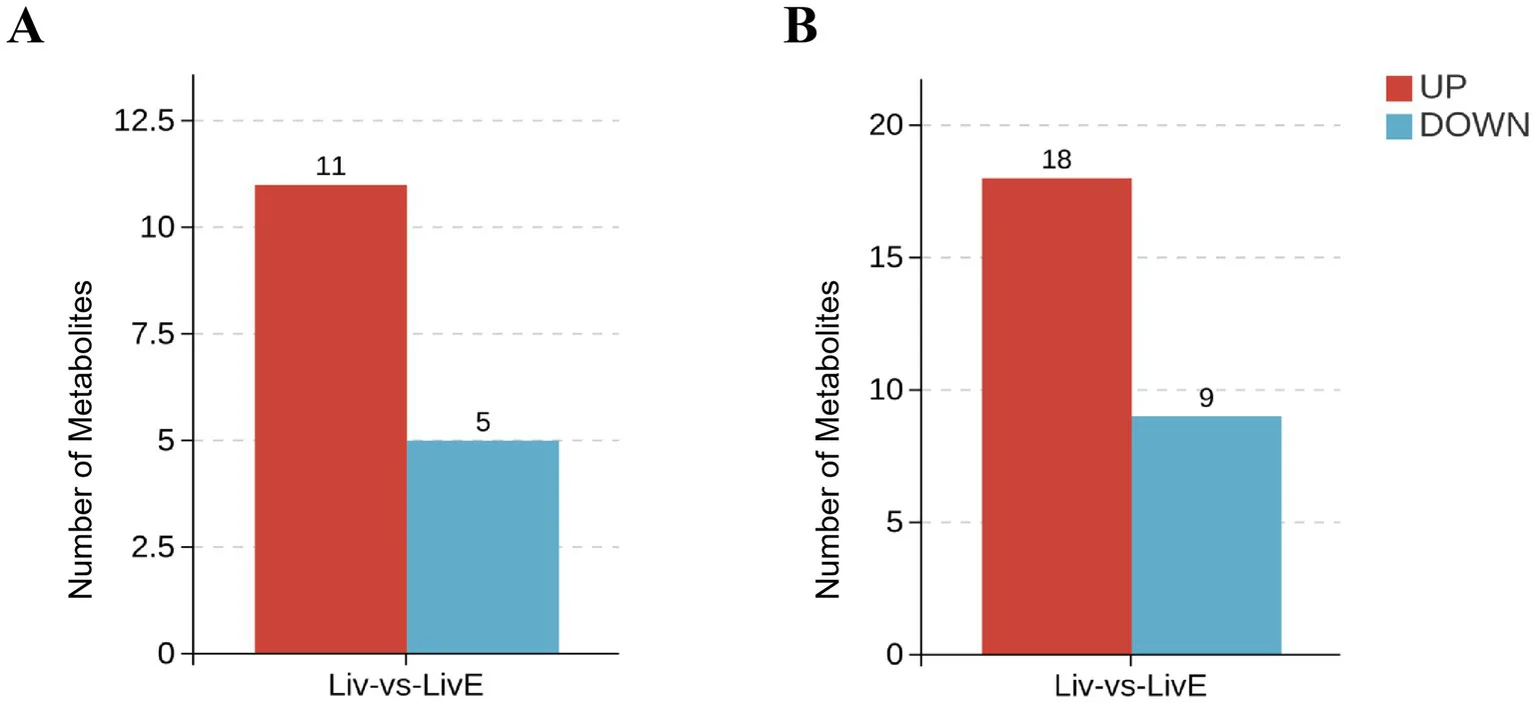
Differences in the expression levels of metabolites in the liver. **(A)** The expression level in positive ion mode. **(B)** The expression level in negative ion mode. Liv represents the control group. LivE represents the *B. coagulans* group.

Metabolites showing differences between two groups were identified by combining VIP values from OPLS-DA with *p*-values from independent samples T-tests (Figure 5). The criteria for significance were: VIP ≥ 1 in the OPLS-DA model and T-test *p* < 0.05. Under the positive and negative ion detection modes, a total of 16 and 27 metabolites with significant concentration differences were identified, respectively. There were 11 upregulated metabolites and 5 downregulated metabolites in positive ion mode, while the negative ion mode group included 18 upregulated metabolites and 9 downregulated metabolites.

**Figure 6:**
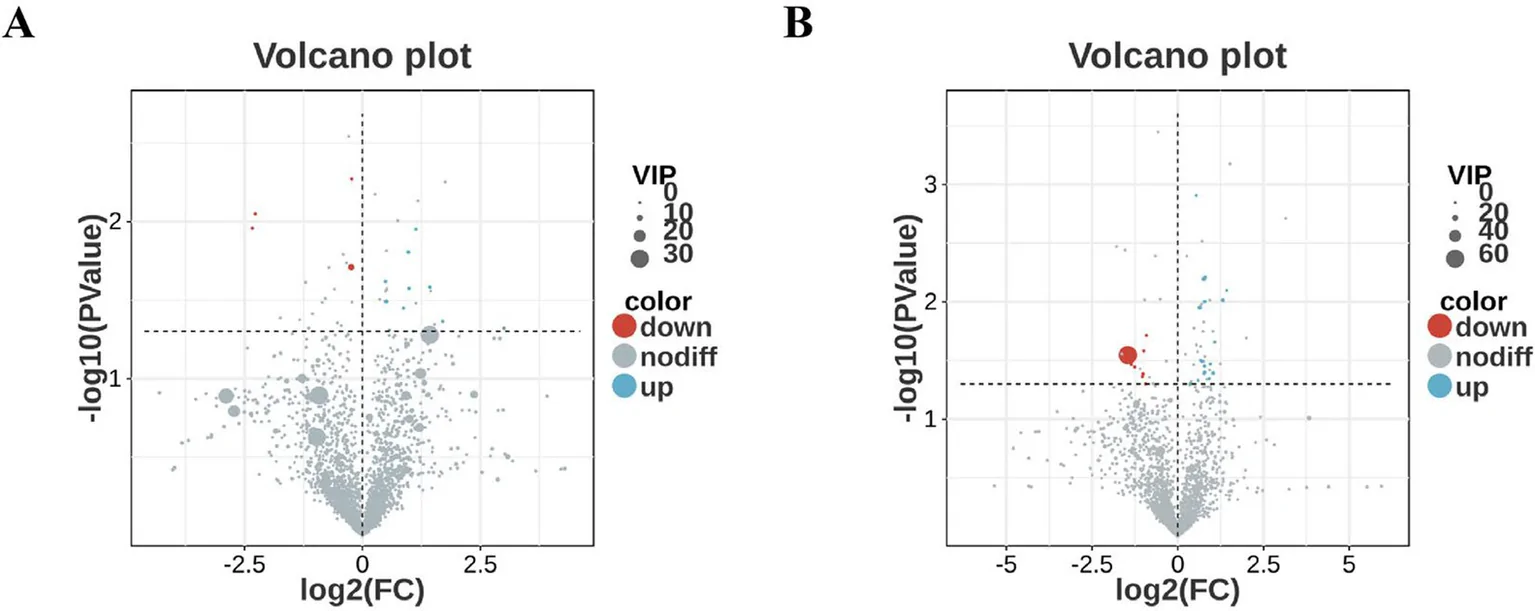
Differentially accumulated metabolites in the liver. **(A)** Volcano plot in positive ion scanning mode. **(B)** Volcano plot in negative ion scanning mode.

To further understand the expression trends of differentially accumulated metabolites (DAMs), fold changes (FC) were calculated. A volcano plot was generated to visualize the significance, fold change, and VI*p* values of the DAMs (Figure 6). The screening criteria were set at *p* < 0.05 with an absolute fold change ≥ 1. The horizontal axis represents the fold change, expressed as log_2_(FC), points closer to either end indicating a larger fold change. The vertical axis represents significance, expressed as -log_10_(*p* value). The horizontal dashed line corresponds to the significance threshold (*p* = 0.05), points closer to the top indicating higher significance. The size of the points depends on their VIP values reflecting the contribution of the metabolite to the OPLS-DA model clustering.

**Figure 7:**
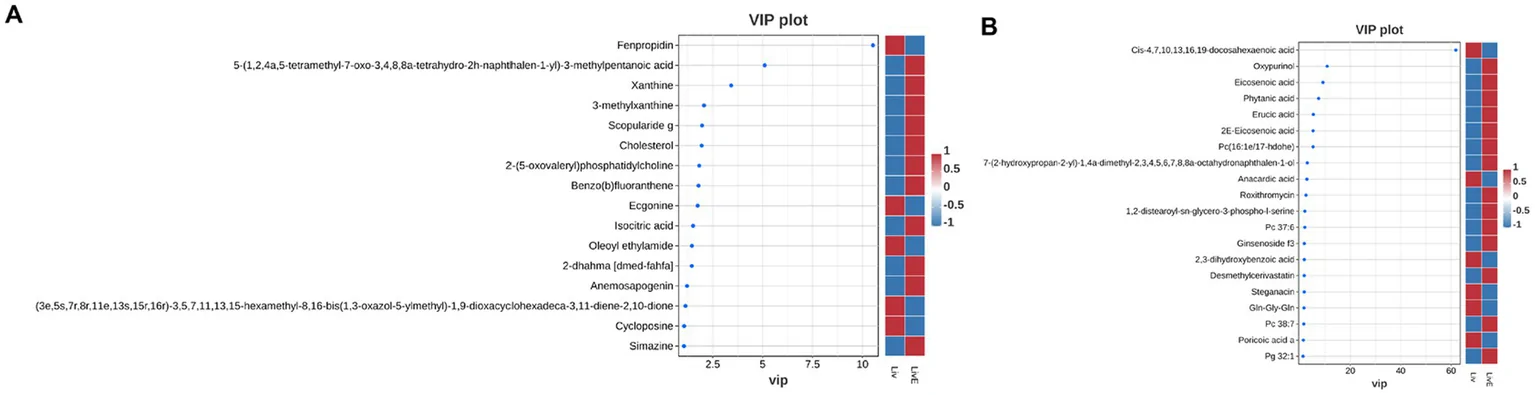
VIP plot of different metabolites in the liver. **(A)** VIP value plot in positive ion mode. **(B)** VIP value plot in negative ion mode. The higher the VIP value, the greater the contribution the metabolite makes to distinguishing samples. By default, metabolites with a VIP > 1 are considered to show a significant difference. VIP: Variable importance in projection.

The VIP results for the top 15 DAMs (at the MS^2^ level) in each group were shown in Figure 7A showed DAMs detected in positive ion mode, with significant changes observed in sterol, carboxylic acid and purine metabolites. Figure 7B showed DAMs detected in negative ion mode, with significant changes observed in fatty acid and allopurinol metabolites.

**Figure 8:**
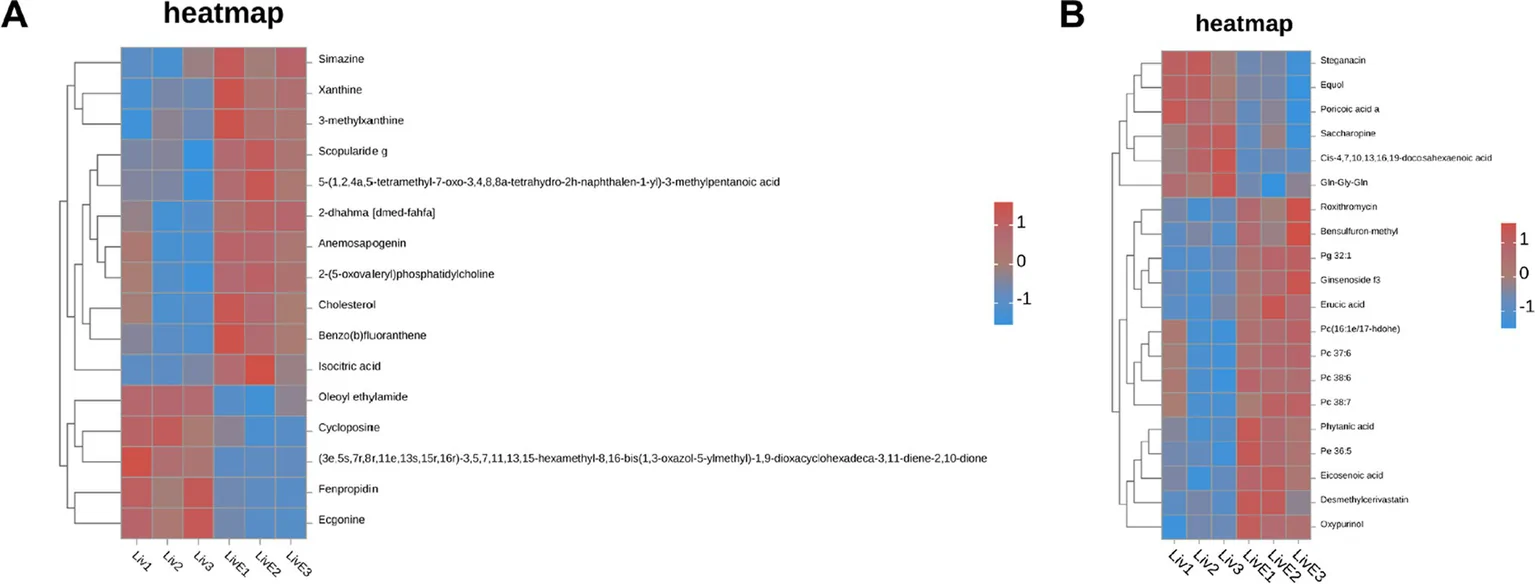
Heatmaps of metabolites expression in the liver. **(A)** Metabolites expression in positive ion mode. **(B)** Metabolites expression in negative ion mode. Red color represents the higher metabolites abundance. Blue color represents the lower metabolites abundance. Liv represents the control group. LivE represents the *B. coagulans* group.

After normalizing the data for DAMs across groups, cluster heatmap analysis was performed (MS^2^ level), which visually illustrated the accumulation differences between groups, with darker colors indicating more pronounced differences. Compared to the control group, the levels of most metabolites were significantly elevated in the *B. coagulans* group, such as purine metabolites, steroid metabolites, and fatty acid metabolites (Figure 8).

#### 3.4.3 KEGG pathway enrichment

**Figure 9:**
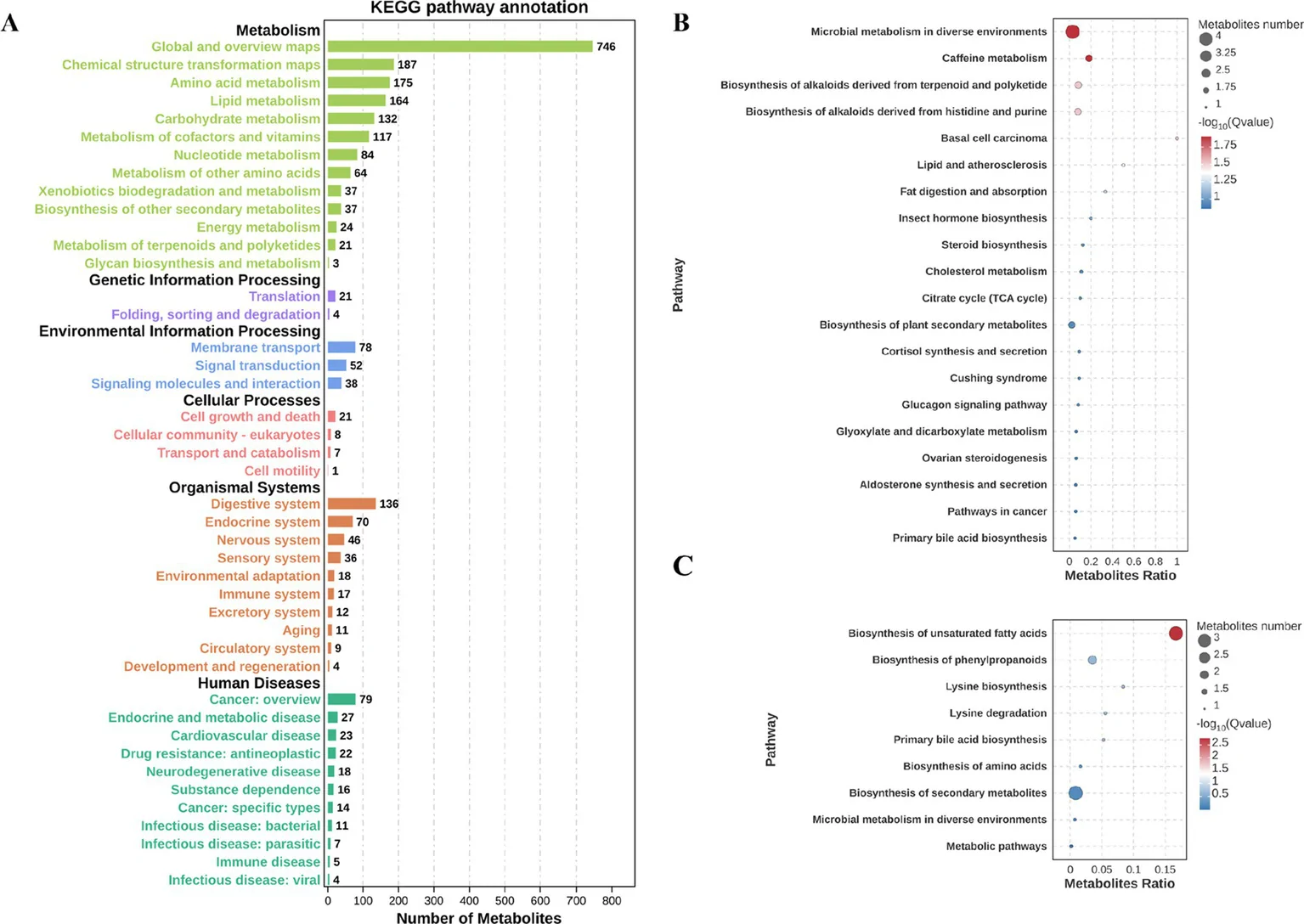
KEGG enrichment analysis of metabolites in the liver. **(A)** KEGG all pathway annotation in the liver. **(B)** KEGG pathway enrichment bubble plot in positive ion mode. **(C)** KEGG pathway enrichment bubble plot in negative ion mode. -Log_10_(*Q* value) represents the significance, the higher the value, the higher the significance.

To further investigate the potential metabolic pathways influenced by *B. coagulans*, pathway enrichment analysis was performed on DAMs using the KEGG database. Figure 9A presented an overview of the KEGG enriched pathways, demonstrating that DAMs were primarily concentrated in pathways such as the global overview, chemical structure transformation, amino acid metabolism, lipid metabolism, and carbohydrate metabolism. Figures 9B,C presented the enriched metabolic pathways under different ion modes. In the positive ion mode, multiple metabolic pathways with significant associations (*p* < 0.05) were identified. In the negative ion mode, the significantly enriched pathway was unsaturated fatty acid biosynthesis.

## 4 Discussion

### 4.1 Effects of *B. coagulans* on hepatic structure and function

The nutritional composition of the basal diet in this study was derived from the manufacturer’s label guarantees, rather than from independent biochemical analyses, which constituted a methodological limitation. Nevertheless, the diet was a standardized commercial product from a single batch, with label values generated through routine quality control procedures and limited batch-to-batch variability. Given that the present study primarily addressed comparative effects, minor absolute deviations in nutrient levels were unlikely to substantially affect the main conclusions.

The liver is the organ in fish responsible for metabolism, detoxification, and blood clotting, and changes in liver morphology indicate health and environmental stress (Li et al., 2022). Khushboo et al. found that after 96 h of exposure to 2 amino benzene sulfonate (2 ABS) which is an intermediate compound in the textile industry, fish liver exhibited varying degrees of toxicological changes, including lymphocyte infiltration, hepatocyte congestion, nuclear condensation, hepatocyte vacuolization, and even necrosis in some areas, leading to the onset of liver disease (Khushboo et al., 2022). Because the liver and intestine are closely connected via the gut-liver axis, probiotics can indirectly influence the liver through the axis. In the study of Chinese perch, Feng et al. found that adding *B. subtilis* BS1 and *Lactobacillus plantarum* LP1 to feed could reduce hepatic lipid deposition, thereby preventing the development of fatty liver disease (Feng et al., 2021). In a similar vein, adding the probiotic SLB51 (a mixture of lactic acid bacteria and bifidobacteria) and yeast-derived prebiotics alleviated liver damage in freshwater fish caused by toxic substances, and even restored the fish to a state close to health (Christian et al., 2023; Fahla et al., 2026). In the present study, the addition of *B. coagulans* resulted in significant improvement in hepatic morphology of juvenile *E. tetradactylum*. The histological sections demonstrated intact structures and orderly cellular arrangement, while pathological changes around the central vein and the degree of fibrosis also exhibited a decreasing trend. The present results demonstrated that *B. coagulans* played a positive role in improving hepatic morphology and alleviating fibrosis and pathological changes.

Large amounts of opportunistic pathogens are constantly present in aquatic environments. When fish experience a decline in immunity, these bacteria can invade the body and cause disease. Alkaline phosphatase (AKP), one of the key indicators for assessing physical health status, participates in various physiological and metabolic activities, such as cell differentiation, the transfer and metabolism of phosphate groups, and the metabolism of other nutrients (Ma et al., 2021). Furthermore, it prevents toxic substances from entering the body and plays an active role in the immune system by enhancing the ability to recognize and phagocytose pathogens. It is crucial for growth, metabolism, homeostasis, and immune function (Ming et al., 2015). Lysozyme (LZM), the primary nonspecific immune mediator, can hydrolyze peptidoglycan bonds in bacterial cell walls while also activate complement and promote phagocytosis, playing a widespread role in resisting parasitic, bacterial, and viral infections (Puangkaew et al., 2004; Patrizia et al., 2021). Together with other immune factors, it participates in the host non-specific immune defense (Rauta et al., 2012). Singh and Pandey found that adding the probiotic *L. plantarum* FLB1 to freshwater fish feed increased LZM activity and enhanced the immunity (Singh and Pandey, 2026). Likewise, *L. acidophilus* and *B. subtilis* added to carp feed were found to result in a significant increase in LZM activity after a period of time (Aly et al., 2008). Qiu et al. found that adding *L. acidophilus* as a probiotic to zebrafish (*Danio rerio*) feed led to a significant increase in AKP and LZM activity in the fish (Qiu et al., 2025). In this study, the addition of probiotic did not result in significant changes in AKP and LZM, suggesting that *B. coagulans* did not exert a significant regulatory effect on AKP and LZM. This finding could be further validated by adjusting the dosage of the probiotic in subsequent studies.

Alanine aminotransferase (ALT) is a crucial enzyme in amino acid metabolism and plays a dominant role in hepatic gluconeogenesis (Ijeoma et al., 2026), elevation of which in serum typically indicates liver damage (Zhang Z. et al., 2025). Inversely, in hepatic tissue, increased ALT activity tends to indicate the hepatic health. When hepatocytes are damaged, intracellular enzymes are allowed to release into the blood, which leads to elevation of ALT level (McGill, 2016). Some assays demonstrated that the increase of ALT activity reflects an enhanced capacity for amino acid catabolism and energy turnover (Brosnan and Brosnan, 2009; Silvia and Carlos, 2012; Pagliarini et al., 2016). Zhang et al. found that dietary vitamin B6 increased ALT activity in the livers of largemouth bass (*Micropterus salmoides*) and promoted amino acid metabolism (Zhang L. et al., 2025). In the present study, used samples were liver tissue rather than serum samples. Coupled with the improved liver morphology observed in the histological examination, the elevation indicated that *B. coagulans* effectively promoted basal hepatic metabolism and nutrient utilization rather than causing tissue injury. This finding was consistent with the enhanced metabolic profiles observed in our metabolomic analysis, suggesting that the probiotic optimizes energy allocation and metabolic efficiency in juvenile *E. tetradactylum*.

Reactive oxygen species (ROS) are metabolites produced during normal physiological activities. When ROS production becomes imbalanced and exceeds the antioxidant defense capacity, oxidative stress occurs, leading to cellular dysfunction or even cell death (Cheng et al., 2019; Cheng et al., 2018). Oxidative stress is also defined as a contributing factor to liver damage. Fish possess endogenous antioxidant systems that limit oxidative damage caused by ROS generated during metabolism, environmental stress, and infection (Islam et al., 2026). The liver contains antioxidants such as SOD and CAT, which maintain the redox balance by clearing excess free radicals (Cheng et al., 2023). MDA is a byproduct of lipid peroxidation caused by ROS attacking polyunsaturated fatty acids in cell membranes and serves as a marker of oxidative stress damage (Antonio et al., 2014). Abdel-Latif et al. found that dietary probiotics supplement to crystal barb fry significantly resulted in increasing CAT and SOD activity while reducing MDA levels (Abdel-Latif et al., 2023). In addition, Linh et al., Aziz et al., and Mathew et al. found that adding probiotics to the diet of Nile tilapia or New Kigali tilapia significantly led to an increase in SOD activity (in a dose-dependent manner in Linh et al.) and CAT activity (in the latter two studies), while MDA levels showed no significant change in Linh et al. but notably reduced in the other two studies (Linh et al., 2026; Aziz et al., 2025; Mathew et al., 2025). In the present study, the levels of MDA, CAT, and SOD in the *B. coagulans* group did not exhibit statistically significant when compared to the control group (*p* > 0.05). This non-significant fluctuation indicated that dietary supplementation with *B. coagulans* safely maintained the hepatic redox homeostasis without inducing oxidative stress. The preservation of baseline antioxidant enzyme activities, coupled with the slight reduction in the lipid peroxidation marker MDA, suggested that the basal oxidative pressure in the liver was well-controlled. It was plausible that the optimized metabolic flux and the enhanced free-radical scavenging capacity driven by key metabolites (such as xanthine and IA, as revealed in our metabolomic analysis) buffered the internal environment, thereby reducing the reliance on the compensatory upregulation of endogenous antioxidant enzymes.

### 4.2 Effects of *B. coagulans* on the metabolomics of juvenile *E. tetradactylum*

*B. coagulans* is commonly mixed into daily feed as a dietary supplement. It enhances immunity and anti-inflammatory and antioxidant capabilities in farmed fish, while also reduce hepatic damage and regulate the balance of the intestinal microbiota, making it widely used in the aquaculture industry (Khalil et al., 2026; Yuan et al., 2026; Zhang Y. et al., 2025). Previous study showed that dietary *B. coagulans* supplementation can enhance the growth of *E. tetradactylum*, reducing feed conversion efficiency while improving digestive absorption. The present study found that *B. coagulans* can also enhance metabolic capacity. Taken together, these findings suggested that *B. coagulans* may first enhance metabolic function, thereby optimizing the growth performance, which provided a new perspective for elucidating its probiotic mechanism (Zheng et al., 2025).

In the present study, an OPLS-DA model was constructed to evaluate the overall metabolic separation between the control group and the *B. coagulans* group. The score plot of the model revealed a clear separation in metabolic profiles between the two groups, indicating systematic metabolic differences. However, in the subsequent permutation test, the Q^2^ values for both positive and negative ion modes were below 0.42, indicating that the model did not achieve satisfactory predictive performance and suggesting a risk of overfitting. This outcome was probably attributable to the insufficient sample size per group (n < 6) for the LC/MS analysis, since a small sample size tended to amplify random noise and thus compromised the robustness and external validity of the statistical model.

It should be noted that OPLS-DA was only a component of the multivariate statistical analyses in this study, and we did not rely solely on this model to draw conclusions. To achieve a more comprehensive and objective screening and validation of differential metabolites, we also employed multiple complementary differential analysis approaches, including hierarchical clustering heatmaps to visualize the grouping consistency of metabolite expression patterns, volcano plots to integrate fold-change and statistical significance. Furthermore, at the functional level, we performed KEGG pathway enrichment analysis to further corroborate the potential physiological significance of the differential metabolites from the perspective of biological pathways.

In summary, although the suboptimal predictive performance of the OPLS-DA model somewhat weakened its direct discriminative power, the results derived from visualized tools and functional enrichment analysis were mutually consistent, collectively forming a multidimensional chain of evidence supporting the credibility of the identified differential metabolites. Therefore, we considered that, under the present experimental conditions, integrating multiple analytical strategies can effectively compensate for the limitations of any single model, and the obtained differential metabolites still provide reliable biological reference value.

A total of 43 differentially accumulated metabolites were identified across both positive and negative ion modes in the *B. coagulans* group. Metabolic pathways significantly altered were identified via pathway enrichment analysis, primarily involving energy metabolism (tricarboxylic acid), lipid metabolism (fat digestion and absorption, cholesterol metabolism, and unsaturated fatty acid biosynthesis), and secondary metabolite synthesis (plant secondary metabolite biosynthesis).

The tricarboxylic acid (TCA) cycle, a series of enzymatic reactions occurring within mitochondria, serves a central pathway in cellular metabolism and a key component of cellular respiration (Kozlova et al., 2026). The present study found that the levels of isocitric acid (IA) and xanthine were significantly elevated in the livers of juvenile *E. tetradactylum*. IA participates in TCA cycle in the form of the isocitrate, reflecting the reaction rate of TCA cycle (Inmaculada and Navdeep, 2020). Catalyzed by isocitrate dehydrogenase, isocitrate is converted into *α*-ketoglutarate, which is a key step TCA cycle. The nicotinamide adenine dinucleotide phosphate (NADPH) can be converted by NADH produced by TCA cycle via proton-translocating transhydrogenases, which is essential for glutathione peroxidase (GPx) scavenging ROS within mitochondria, thereby alleviating oxidative damage to cells (Pedersen et al., 2008; Lee et al., 2020).

Xanthine is a purine metabolic intermediate that exhibits potent free-radical scavenging capabilities under healthy physiological conditions. Although its conversion to uric acid by xanthine oxidase can generate reactive oxygen species during severe stress, its primary function in an optimized hepatic environment is to act as an endogenous antioxidant by inhibiting the oxidative cleavage of hydroxyl radicals (Sonish et al., 2003; Althaher et al., 2026; Meng et al., 2026).

In this study, the upregulation of xanthine, alongside IA, did not induce oxidative stress. On contrary, it synergistically enhanced the capacity to scavenge ROS of liver. This accelerated purine turnover facilitated the rapid clearance of metabolic waste, exerting a robust cytoprotective effect and indicating that *B. coagulans* positively contributed to regulate hepatic metabolic homeostasis.

Lipid metabolism is considered as a key regulatory pathway in aquatic animals under environmental stress, providing essential fatty acids and energy while supporting immune responses (Fernando et al., 2013). The liver is a key organ in lipid metabolism, and cholesterol is a key substance in this process. It serves as a precursor for the synthesis of various essential compounds and plays multiple physiological roles in metabolic processes (Shen et al., 2026). When cholesterol combines with taurine or glycine, bile acids are formed, which in turn promote the emulsification and absorption of fats in the intestine (Fuchs et al., 2024). Wu et al. found that the expression of fatty acid synthase mRNA of grass carp *(Ctenopharyngodon idella)* were reduced by dietary *B. subtilis* supplement while genes related to fatty acid *β*-oxidation showed an upregulated trend, indicating that the probiotic played a dual role in fish. On one hand, it can inhibit lipid synthesis and reduce fat production. On the other hand, it actively promotes lipid catabolism, thereby exerting a profound influence on the lipid metabolic balance and improving hepatic lipid metabolism (Wu et al., 2023). When dietary cholesterol levels are low, fish have to rely on endogenous cholesterol synthesis, which may increase energy expenditure and affect growth performance (Fernando et al., 2013). In summary, fish maintain cellular cholesterol homeostasis through the precise regulation of processes such as cholesterol synthesis, uptake, transport, excretion, and esterification. In this study, while lipid metabolism was identified in the enriched pathway, increasing cholesterol levels enhanced hepatic metabolic capacity, thereby promoting cholesterol metabolism and excretion, indicating that *B. coagulans* played a positive role in hepatic health.

## 5 Conclusion

In conclusion, dietary supplementation with *B. coagulans* was found to improve hepatic tissue morphology and optimized key metabolic processes, particularly amino acid turnover and lipid metabolism in juvenile *E. tetradactylum*. While baseline immune and antioxidant enzyme activities were largely maintained, the probiotic was capable to alleviate lipid peroxidation and oxidative damage. These findings demonstrated its substantial potential as a functional additive for promoting hepatic health and metabolic homeostasis in aquaculture.

## Data Availability

The raw data supporting the conclusions of this article will be made available by the authors, without undue reservation.
