# Correction: Dietary *Bacillus coagulans* supplementation improves liver health and modulates hepatic metabolic profiles in juvenile fourfinger threadfin (*Eleutheronema tetradactylum*)

**DOI:** 10.3389/fmicb.2026.1982030

**Published:** 2026-09-17

**Authors:** Jing Li, Nuo Chen, An-Na Zheng, Shui-Ping He, Wei-Bin Liu, Jing-Heng Lu, Min-Xuan Jin, Lin-Juan Wang, Hui-Juan Zhang, Evodia Moses Mkulo, Bao-Gui Tang, Hui Zhou, Bei Wang, Jian-Sheng Huang, Zhong-Liang Wang

**Affiliations:** 1 College of Fisheries, Guangdong Ocean University, Zhanjiang, Guangdong, China; 2 Guangdong Marine Fish Science and Technology Innovation Center, Zhanjiang, Guangdong, China; 3 Guangdong Provincial Key Laboratory of Aquatic Animal Disease Control and Healthy Culture, Zhanjiang, Guangdong, China; 4 Agro-Tech Extension Center of Guangdong Province, Guangzhou, China

**Keywords:** *Bacillus coagulans*, *Eleutheronema tetradactylum*, hepatic metabolism, metabolomics, probiotics

An incorrect number was provided for the funder “Research on breeding technology of candidate species for Guangdong modern marine ranching”. This was originally given as (2024-MRB-00-001). The correct number is (2025-MRB-00-001). The correct funding statement should be read as follows: “Funding: The author(s) declared that financial support was received for this work and/or its publication. This research was funded by the 2025 Research on breeding technology of candidate species for Guangdong modern marine ranching (2025-MRB-00-001), the Guangdong Province Ordinary Colleges and Universities Key Field Special Project (Science and Technology Services for Rural Revitalization) (2023ZDZX4011), and the 2026 Zhanjiang 'Talent Revitalization Plan for the Modern Marine Ranching Industry' Project.”

The original version of this article has been updated.

